# Mediastinal Extramedullary Plasmacytoma in the Absence of Classical Myeloma Markers: A Case Report

**DOI:** 10.7759/cureus.99530

**Published:** 2025-12-18

**Authors:** Gonçalo Torres, Ana Patrícia Brito, Hermínia Teixeira, Jorge Cotter, Paula Mota

**Affiliations:** 1 Clinical Pathology, Unidade Local de Saúde do Alto Ave, Guimarães, PRT; 2 Internal Medicine, Unidade Local de Saúde do Alto Ave, Guimarães, PRT

**Keywords:** atypical myeloma presentation, extramedullary plasmacytoma, free light chains, light chain multiple myeloma, mediastinal mass, pleural effusion

## Abstract

Multiple myeloma (MM) typically presents with bone pain, pathological fractures, anemia, renal impairment, or detectable monoclonal gammopathy. In rare instances, MM may manifest with extramedullary plasmacytomas, and mediastinal involvement can challenge diagnostic expectations.

We report the case of a 69-year-old man presenting with severe shoulder pain and progressive dyspnea who was found to have a large anterior mediastinal mass, bilateral pleural effusion, and multiple bone lesions. Routine laboratory evaluation revealed no anemia, renal dysfunction, or detectable monoclonal protein on serum protein electrophoresis. However, serum-free light chain assay revealed markedly elevated lambda chains. Biopsy confirmed plasmacytoma. The final diagnosis was light chain MM with mediastinal extramedullary plasmacytoma.

This case underscores the importance of considering plasmacytoma in the differential diagnosis of mediastinal masses, even when classical laboratory markers of myeloma are absent, and highlights the pivotal role of histopathology and advanced laboratory testing in establishing a timely diagnosis.

## Introduction

Multiple myeloma (MM) accounts for approximately 1% of all malignancies and about 10% of hematologic cancers [1]. While classically characterized by bone pain, anemia, renal dysfunction, and a detectable monoclonal protein, MM can also present with extramedullary involvement, where clonal plasma cells proliferate outside the bone marrow microenvironment. Extramedullary plasmacytomas (EMPs) occur in fewer than 5% of MM cases [2], yet their clinical significance greatly exceeds their rarity. This may occur either as a solitary EMP (primary EMP) or, more commonly, as extramedullary disease secondary to systemic MM, the latter being associated with more aggressive biological behavior and poorer prognosis [3].

Although EMP constitutes only a small proportion of MM manifestations, its clinical relevance is substantial. Mediastinal involvement, in particular, is exceptionally rare, with only isolated cases documented, and is often linked to advanced disease dissemination and therapeutic resistance [3].

This case is noteworthy for its unusual mediastinal presentation and the absence of classical myeloma-defining laboratory abnormalities, including a completely normal serum protein electrophoresis (SPE). Such presentations pose significant diagnostic challenges, as light chain-only disease may remain undetected without targeted testing. Few published reports describe mediastinal plasmacytoma with normal SPE at diagnosis.

This case highlights the diagnostic pitfalls of light chain MM presenting with EMP and underscores the critical role of serum-free light chain (FLC) assay and tissue biopsy in identifying clinically significant disease that may otherwise be missed.

## Case presentation

A 69-year-old man with an Eastern Cooperative Oncology Group (ECOG) performance status of 0 and no relevant medical history presented to the emergency department with severe left shoulder pain persisting for two weeks, unresponsive to nonsteroidal anti-inflammatory drugs. He also reported progressive dyspnea over the preceding month, which had worsened in recent days.

On physical examination, he was slightly tachypneic (respiratory rate 21 breaths per minute) but was afebrile, hemodynamically stable, and exhibited no signs of respiratory distress. Cardiovascular auscultation revealed normal heart sounds without murmurs or rubs. Pulmonary examination showed preserved breath sounds bilaterally, without wheezes or crackles. The abdomen was soft and non-tender, with no hepatosplenomegaly. No peripheral edema, lymphadenopathy, or cutaneous lesions were noted. Neurological examination was normal.

Initial laboratory evaluation revealed no anemia, normal renal function, no electrolyte disturbances, and blood gas analysis combined with further lab tests showed no significant findings (Table 1).

**Table 1:** Admission laboratory test results. LDH: lactate dehydrogenase

Test description	Result	Reference range
Hemoglobin (g/dL)	15.0	14.0-18.0
Leukocytes (per µL)	7.7 x 10^3	4.8-10.8 x 10^3
Neutrophils (per µL)	4.5 x 10^3	1.8-7.7 x 10^3
Eosinophils (per µL)	0.2 x 10^3	0.0-0.5 x 10^3
Basophils (per µL)	0.1 x 10^3	0.0-0.1 x 10^3
Lymphocytes (per µL)	2.2 x 10^3	1.0-4.8 x 10^3
Monocytes (per µL)	0.7 x 10^3	0.1-0.8 x 10^3
Platelets (per µL)	227 x 10^3	150-350 x 10^3
C-reactive protein (mg/L)	5.4	<3.0
Urea (mg/dL)	37	15-39
Creatinine (mg/dL)	0.9	0.8-1.3
Sodium (mEq/liter)	145	135-146
Potassium (mEq/liter)	4.4	3.5-5.1
Calcium (mg/dL)	9.4	8.3-10.6
Phosphorus (mg/dL)	4.8	2.5-4.9
LDH (UI/liter)	240	120-246
Albumin (g/dL)	4.0	3.4-5.0

**Figure 1:**
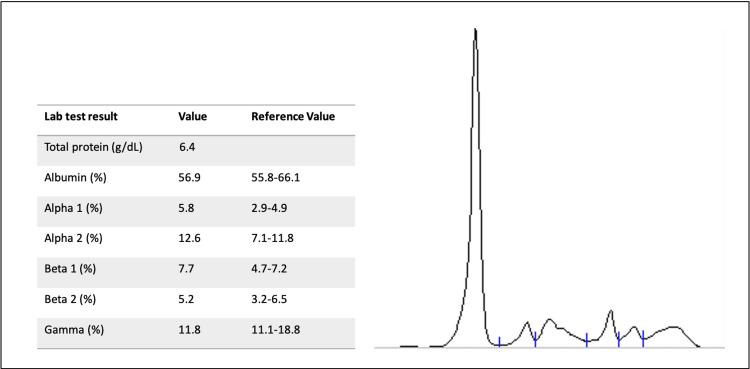
Serum protein electrophoresis demonstrating a normal electrophoretic pattern without a monoclonal spike, consistent with the absence of detectable intact immunoglobulin paraproteins.

SPE was unremarkable, with no monoclonal band or hypogammaglobulinemia (Figure 1).

**Figure 2:**
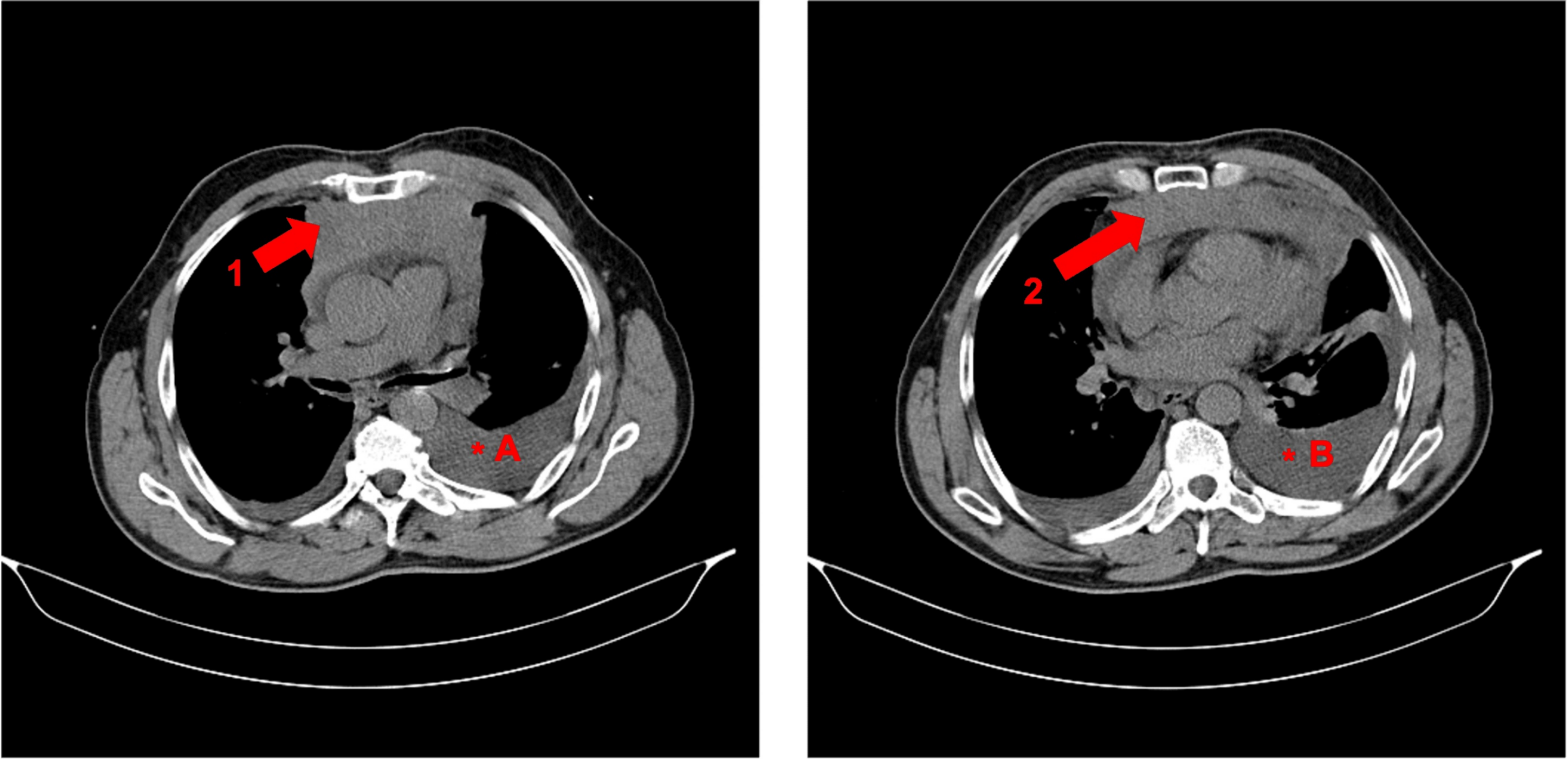
Axial chest CT scan (soft-tissue/mediastinal window) demonstrating a 12.4 × 3.2 cm anterior mediastinal mass. Red arrows (1) and (2) indicate the anterior mediastinal mass, while asterisks (*A and *B) denote pleural effusions. CT: computed tomography

Given the symptoms, chest radiography was performed and revealed an enlarged cardiac silhouette, rightward tracheal deviation, and a right pleural effusion. These findings were further evaluated through a chest computed tomography (CT), which revealed a solid, expansive anterior mediastinal mass measuring 12.4 × 3.2 cm (Figure 2), associated with bilateral pleural effusion (larger on the left) leading to atelectasis, a hypodense lesion in the T10 vertebra, and small intra-abdominal lymph nodes (<1 cm).

Subsequent echocardiography revealed no pericardial effusion or other abnormalities.

**Figure 3:**
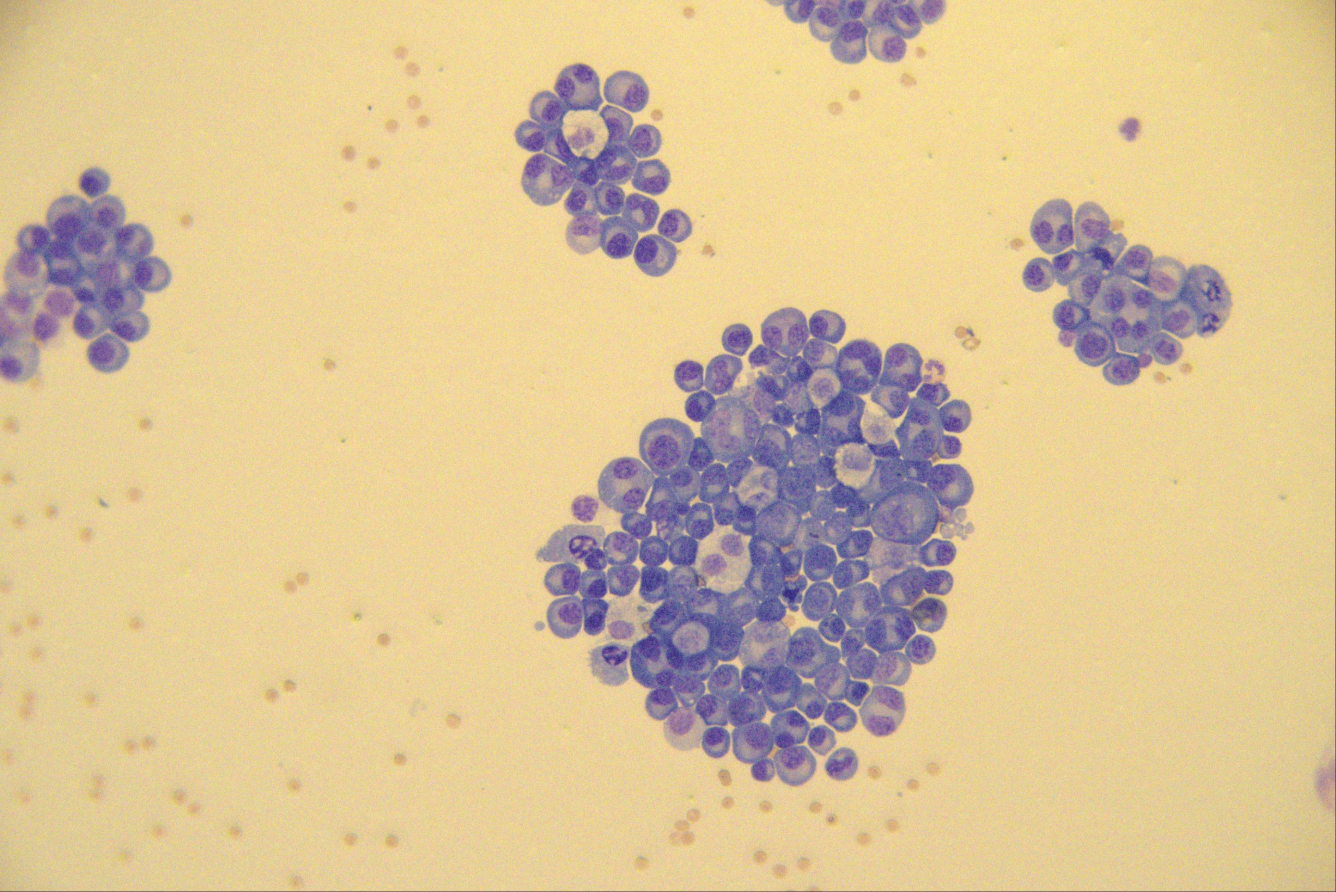
Pleural fluid cytospin preparation (May-Grünwald stain, ×400 magnification) showing numerous plasma cells, including multinucleated atypical forms, supporting a plasma-cell neoplasm.

Diagnostic thoracentesis was performed on the same day. Pleural fluid analysis demonstrated an exudate with 93% mononuclear cells and 3,600 nucleated cells/µL. Pleural fluid cytospin showed many mononuclear plasma cells (both mature and immature forms), some of which presented dysplastic features, such as multiple nuclei (Figure 3).

Given these findings, a serum-FLC assay was obtained on day 1 and revealed markedly elevated lambda FLCs (5520 mg/L; upper limit of normal 27 mg/L), with a kappa/lambda ratio <0.001.

**Figure 4:**
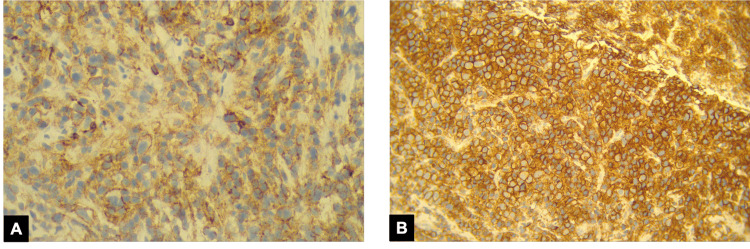
Immunohistochemistry of the mediastinal mass biopsy. (A) CD56 positive immunostaining highlighting aberrant expression in neoplastic plasma cells. (B) CD138 immunostaining demonstrating strong cytoplasmic positivity typical of plasma-cell lineage.

On hospital day 2, a CT-guided biopsy of the mediastinal mass revealed infiltration by atypical plasma cells, positive for CD138 and CD56 on immunohistochemistry, consistent with plasmacytoma (Figure 4).

Subsequent serum immunoglobulin quantification performed on day 4 showed IgG 1120 mg/dL (reference 700-1600), IgA 215 mg/dL (reference 70-400), IgM 74 mg/dL (reference 40-230), and β2-microglobulin 1.8 mg/L (reference 1.1-2.4), all within normal ranges, further supporting the absence of systemic myeloma involvement.

**Figure 5:**
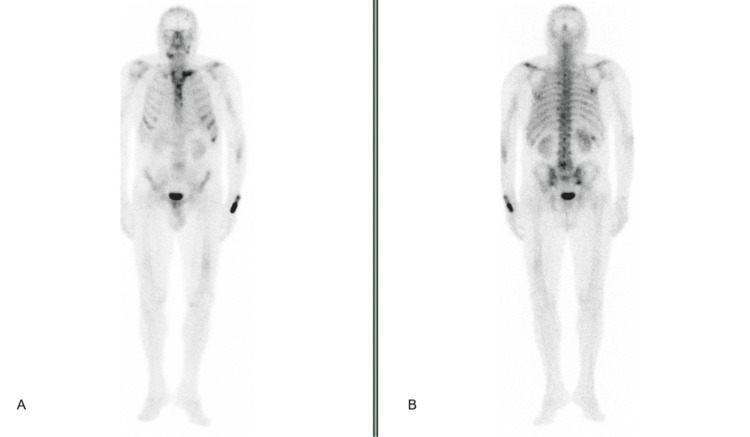
Whole-body bone scintigraphy showing (A) anterior and (B) posterior views, demonstrating multiple areas of increased radiotracer uptake involving axial and appendicular skeleton, consistent with widespread lytic lesions.

On day 7, bone scintigraphy demonstrated multiple lytic bone lesions, including the axial skeleton, skull, left femur, clavicles, scapulae, and ribs (Figure 5).

Given the presence of a mediastinal mass in a patient without classic myeloma-defining laboratory abnormalities, initial differential diagnoses included thymoma, lymphoma, germ cell tumor, metastatic carcinoma, and granulomatous disease. The absence of serum markers suggestive of germ cell neoplasms and the histological and immunophenotypic profile favoring plasmacytic differentiation progressively narrowed the differential diagnosis. Only through progressive clinical reasoning and systematic exclusion, supported by imaging and ultimately by histopathology, was the true nature of the lesion established.

The final diagnosis was light chain MM with mediastinal EMP and multiple lytic bone lesions. The patient was transferred to a regional oncology center for comprehensive staging and initiation of systemic therapy.

## Discussion

EMPs are rare manifestations of MM, and mediastinal involvement represents one of the least common presentations, often leading to diagnostic delay because symptoms such as dyspnea and shoulder pain are nonspecific and easily attributed to musculoskeletal or thoracic conditions [3]. In this case, the unusual anatomical localization combined with the absence of classical laboratory abnormalities significantly complicated the initial diagnostic approach.

Diagnosing extramedullary MM requires the integration of imaging characteristics, cytology, immunohistochemistry, and targeted laboratory studies. Although SPE is a cornerstone test in the initial workup of plasma cell dyscrasias, its diagnostic sensitivity is substantially reduced in light chain-only disease. SPE fails to detect monoclonal FLCs because these low-molecular-weight proteins are not readily visible as a distinct M-spike on electrophoretic tracing. This explains why SPE was normal in our patient despite a heavy tumor burden. In contrast, the serum-FLC assay directly quantifies unbound kappa and lambda chains and therefore plays a critical role in identifying cases of light chain MM that would otherwise remain undetected. The markedly elevated lambda FLC and profoundly abnormal ratio were essential in raising suspicion for an underlying plasma cell neoplasm.

The imaging findings also contributed to diagnostic refinement. A large anterior mediastinal mass with multifocal skeletal involvement is atypical for most thoracic malignancies but aligns with patterns described in disseminated extramedullary MM. The multiplicity of lytic lesions on bone scintigraphy further supported systemic disease rather than a solitary plasmacytoma or alternative mediastinal tumor. Differential diagnoses, including thymoma, lymphoma, germ-cell tumor, and metastatic carcinoma, were considered; however, the immunophenotype (CD138 and CD56 positivity) and plasma cell morphology were incompatible with these entities, and no clinical or radiologic evidence suggested an alternative primary malignancy.

Biologically, extramedullary involvement is believed to reflect altered plasma cell adhesion and homing mechanisms, enabling tumor cells to proliferate independently of the bone marrow microenvironment. This migration capacity is associated with increased therapeutic resistance and poorer outcomes [4]. The extremely high lambda FLC level in our case may further indicate an aggressive secretory phenotype and higher disease burden, factors that often prompt early initiation of systemic therapy. Although radiotherapy can be used for local control, disseminated extramedullary disease typically requires MM-directed systemic chemotherapy [5].

This case underscores several key diagnostic principles: MM cannot be excluded by a normal SPE, imaging findings consistent with disseminated skeletal involvement should heighten suspicion for systemic disease, and extramedullary presentations, particularly in the mediastinum, must prompt early FLC testing and tissue biopsy. A multidisciplinary approach involving pathology, radiology, hematology, and oncology is essential to achieve timely diagnosis and initiate treatment without delay.

Advanced staging and prognostic studies - including bone marrow biopsy, urine immunofixation, cytogenetic and fluorescence in situ hybridization (FISH), Ki-67/MIB-1 proliferation index, and assessment of light-chain restriction by flow cytometry - were not completed at the referring hospital because the patient was transferred promptly to a regional oncology center, where full diagnostic workup and therapeutic planning were undertaken. These investigations, typically necessary to establish definitive staging and risk stratification, therefore fall outside the scope of this case report, which focuses specifically on the diagnostic challenge encountered at initial presentation and the critical role of serum-FLC and tissue biopsy in recognizing an atypical form of MM.

## Conclusions

This case illustrates how MM may first manifest through an extramedullary mediastinal plasmacytoma, even in the absence of classical laboratory abnormalities such as anemia, renal impairment, hypercalcemia, or a detectable monoclonal band on serum electrophoresis. The normal SPE in this patient underscores a key diagnostic pitfall: light-chain-predominant disease may go unrecognized unless serum FLC testing is performed early. The markedly elevated lambda FLC concentration, together with histopathologic confirmation of plasmacytoma and the presence of multiple lytic bone lesions, strongly supported a presumptive diagnosis of light-chain MM with extramedullary involvement, acknowledging that full staging - including bone marrow biopsy, urine studies, and cytogenetic profiling - was deferred to the regional oncology center and therefore lies outside the scope of this report.

From a clinical standpoint, this case reinforces three practical lessons: (1) unexplained mediastinal masses should include plasmacytoma in the differential diagnosis; (2) normal serum electrophoresis does not exclude clinically significant plasma-cell neoplasia; and (3) biopsy and extended laboratory evaluation, particularly serum-FLC assays, are essential when imaging suggests systemic disease. Ultimately, this presentation highlights the need for diagnostic vigilance and early multidisciplinary coordination to ensure timely recognition and management of atypical and potentially aggressive plasma-cell disorders.
